# Rehabilitation in Older Adults Affected by Immobility Syndrome, Aided by Virtual Reality Technology: A Narrative Review

**DOI:** 10.3390/jcm12175675

**Published:** 2023-08-31

**Authors:** Marek Zak, Magdalena Wasik, Tomasz Sikorski, Krzysztof Aleksandrowicz, Renata Miszczuk, Daniel Courteix, Frederic Dutheil, Aneta Januszko-Szakiel, Waldemar Brola

**Affiliations:** 1 Institute of Health Sciences, Collegium Medicum, Jan Kochanowski University, Zeromskiego 5, 25-369 Kielce, Poland; wbrola@wp.pl; 2 Doctoral School, Collegium Medicum, Jan Kochanowski University, Zeromskiego 5, 25-369 Kielce, Poland; magdalena.wasik@phd.ujk.edu.pl (M.W.); tomasz.sikorski@phd.ujk.edu.pl (T.S.); 3 Department of Physiotherapy, Faculty of Health Sciences, Wroclaw Medical University, T. Chałubińskiego 3, 50-368 Wroclaw, Poland; krzysztof.aleksandrowicz@umw.edu.pl; 4 Institute of Heart Diseases, University Hospital, Borowska 213, 50-556 Wroclaw, Poland; 5 Institute of Pedagogy, Jan Kochanowski University, Zeromskiego 5, 25-369 Kielce, Poland; rmiszczuk@ujk.edu.pl; 6 Laboratory of the Metabolic Adaptations to Exercise under Physiological and Pathological Conditions (AME2P), Université Clermont Auvergne, 63000 Clermont-Ferrand, France; daniel.courteix@uca.fr; 7 Occupational and Environmental Medicine, CHU, 63000 Clermont-Ferrand, France; fdutheil@chu-clermontferrand.fr; 8 Physiological and Psychosocial Stress, Université Clermont Auvergne, CNRS, LaPSCo, 63000 Clermont-Ferrand, France; 9 Institute of Information Studies, Faculty of Managment and Social Communication, Jagiellonian University, ul. Lojasiewicza 4, 30-348 Krakow, Poland; aneta.januszko-szakiel@uj.edu.pl

**Keywords:** rehabilitation strategies, virtual reality technology, immobility syndrome, older adults, falls prevention

## Abstract

Individual mobility deficit in older adults induces a variety of medical conditions, diminishing their functional capacity in pursuing activities of daily living. In immobility syndrome patients, such conditions are prone further deterioration through a drastically reduced scope of physical activity, owing mostly to poor self-motivation and the monotonous character of conventional rehabilitation regimens. As evidenced by published research, virtual reality technology solutions in rehabilitation management actually add significantly to patients’ self-motivation, while promoting their active involvement in therapy through visual, auditory, and kinaesthetic stimuli. Effective rehabilitation training aided by virtual reality solutions helps patients acquire specific physical and cognitive skills to be subsequently emulated in the real-world environment. The extra added advantage lies in facilitating such training within patients’ own home environments, combined with online monitoring of their progress, when not personally supervised by a physiotherapist, which also boosts the overall cost effectiveness of the therapeutic management itself. This narrative review appears to be the very first one principally focused on critically comparing individual immobilisation with immobility syndrome, especially through the application of the Authors’ own substantial hands-on therapeutic experience in managing various rehabilitation schemes, specifically aided by diverse virtual reality technology solutions.

## 1. Introduction

Technological advances, including virtual reality (VR), are increasingly being used in rehabilitation. VR is based on creating an artificial reality by making creative use of advanced information technology (IT) solutions [1,2]. The key working premise of rehabilitation aided by VR consists of availing a patient of a unique opportunity to acquire specific skills that subsequently may well be used in reality through the selection of appropriate exercises [3].

Individuals affected by immobility syndrome (IS) are bound to have their disorder deteriorate further through a drastically reduced scope of physical activity, owing mostly to lack of any self-motivation, compounded by a sense of chronic fatigue. One of the widely acknowledged contributing factors is that effective conventional training methods are also regarded by some patients as being overly monotonous and fatigue inducing.

On the other hand, a readily available body of research on making effective use of VR technology offers good grounds to believe that when applied throughout the process of comprehensive rehabilitation, VR can actually add significantly to patients’ self-motivation and active involvement in therapeutic management through the visual, auditory, and kinaesthetic stimuli routinely relied upon in VR technology [4,5,6].

Individuals admit to extra high levels of focus when carrying out their tasks, as well as a great sense of enjoyment throughout. An individually tailored therapeutic programme, making creative use of VR for patients affected by IS, should be effectively harnessed to give older adults extra self motivation, particularly those who seem to be adversely disposed towards pursuing a conventional model of physical activity [7,8,9,10].

Individual mobility deficit is a common enough problem encountered among older adults, and may well be instrumental in the onset of a variety of medical conditions, as well as resulting in an appreciable reduction in functional capacity and ability to perform the routine activities of daily living. Numerous researchers and clinicians alike have attempted to define IS and individual mobility deficit in terms of immobility, but no clear distinction between the two concepts has been agreed upon to date. Most commonly, one’s immobility is construed as being synonymous with individual immobilisation resulting from having been bedridden for an extensive period, for instance due to a serious injury or illness [11,12], or when freely moving about or shifting one’s own body is severely restricted, or simply not feasible at all, for medical reasons [13].

Mobility deficit may also induce the onset of IS in older adults, which is characterised by staying in bed most of the time, although not in response to any specific medical advice, remaining under pharmacotherapy (which actually requires staying in bed), having one’s movement restricted by various orthopaedic devices, consequently being prevented from attempting any movement out of bed without the assistance of a third party, or having been altogether stripped of any self-motivation whatsoever to do so, unless prompted by some external stimuli, for example strong personal persuasion [14].

IS, owing to its prevalence in individuals over 65 years of age, along with its consequences, constitutes one of the Great Geriatric Problems (Geriatric Giants), being also strongly linked, inter alia, to appreciable individual exposure to fall risk [15,16,17]. Additionally, in line with the International Classification of Diseases (ICD-10), paraplegic immobilisation has been established as a concomitant symptom in patients already affected by a frailty syndrome or neurological conditions (M62.3) (ICD-10 2021). As asserted by numerous studies, approximately 50% of patients remain unaware of being affected either by a significant mobility deficit or by IS [18].

VR technology is increasingly being used in post-immobilisation and neurorehabilitation programmes, especially in post-stroke individuals and those that have sustained any form of cranio-cerebral trauma, or those affected by Duchenne muscular dystrophy, multiple sclerosis, Parkinson’s disease, cerebellar ataxia or cognitive impairment [19]. The overall versatility of VR technology as a therapeutic tool of choice is perfectly suited to stimulating patients in many areas of their functional performance, for example, balance, gait, cognitive function, visuospatial abilities, upper limb motor functions (as in post-stroke therapy), and precision movements of the upper limb, including the palm, although by no means does this exhaust the entire scope of its potential applications.

Appreciably enhancing select parameters of movement in older adults is crucial for their functional independence in carrying out the activities of daily living (ADL), and in reducing their exposure to overall risk of falls, both with regard to the individuals affected by IS, and in immobilised ones [20,21,22,23,24]. The application of VR technology in the rehabilitation of previously immobilised individuals also boasts the added advantage of offering the opportunity for a home-based therapy, as well as for monitoring the patient’s progress remotely [24]. This consequently translates into relieving the national healthcare system from this burden, which has been acknowledged to hamper its overall effectiveness.

On the other hand, a thorough medical check-up is essential for patients prior to commencing therapy aided by VR technology (especially with regard to patients immobilised due to neurological dysfunctions), as some studies have indicated that both dizziness and headaches may occasionally be encountered as unwelcome side effects [22].

The aim of this narrative review consists of critically assessing the differences and similarities between immobilisation and immobility in older adults, as well as in highlighting any specific interrelationships between them, while drawing extensively on our own hands-on experience and body of published research. The present review offers practical advice to any clinicians keenly interested in pursuing therapeutic management, specifically aided by select VR technology solutions, in older adults affected by immobility syndrome.

## 2. Materials and Methods

### Search Strategy

This narrative review was based on the search of several databases available online, i.e., PubMed, Google Scholar, EBSCO and Medline. The search, covering an entire decade (since January 2012), was completed in July 2022. Its principal focus rested on individuals over 65 years of age. The search strategy was based on selecting papers reporting both randomised and non-randomised clinical trials that addressed fall risk in older adults affected by immobility syndrome, in due consideration of a feasible therapeutic management that could be applied in such patients, inclusive of selected virtual reality solutions integrated into a specific therapeutic regimen. The following search string was used: older adults OR elderly OR seniors AND (immobility syndrome OR immobilisation) AND (virtual reality OR rehabilitation). The investigators drafted an initial list of articles, which were subsequently reviewed by the Project Manager for potential inclusion and exclusion against clearly defined criteria. Studies were subject to exclusion if they failed to involve older adults or persons affected by IS or immobilisation, or when no rehabilitation regimen was applied in those individuals whatsoever.

## 3. Results

### 3.1. Epidemiology of Individual Immobilisation and IS

Individual risk of IS is widely acknowledged to increase with age, owing to the very nature of the aging process itself, which tends to induce numerous bodily changes over time in all older adults. Studies indicate these changes to be further exacerbated by periods of forced immobility, which is acknowledged to cause appreciable damage to the immobilised parts of one’s body. Additionally, this promotes further disorders in other parts of the body, as if by way of a knock-on effect, thus affecting it as a whole [25]. IS affecting older adults is not so much owing to the incidence of specific diseases, but is rather primarily a direct consequence of significantly reduced physical activity during the daytime, either owing to general weakness of the body itself, or to an individually experienced fear of falling.

Functional limitations experienced by older adults, for example serious difficulties when bending or kneeling (as frequently reported by the patients themselves), account for their preference for spending time in static postures, consequently putting themselves at risk of IS, and appreciably reduced overall quality of life [26,27,28,29]. Such individuals experience a progressive decline in their ability to carry out the routine activities of daily living, as assessed by, for example, the ADL scale [30]. Approximately 20% of individuals over 65 years of age experience significant mobility problems, unless making use of walking aids, or being directly assisted by a third party.

Lack of recommended physical activity adversely affects the hippocampus, causing memory problems in older adults. This may manifest itself, for instance, in the anxiety of forgetting one’s way to a local grocery shop, which may then transform itself into a fear of leaving the house altogether, which would then be bound to evolve into a significant drop in one’s activity during daytime [31]. When over the age of 75, more than half of older adults experience difficulties leaving their apartment, whereas approximately 20% opt to not venture outside at all. The scale of the problem is further highlighted by the estimate that approximately 50% of individuals affected by severe IS are likely to die within the following 6–12 months [32].

The risk of IS varies, depending on the concomitant chronic medical conditions. Studies indicate that with regard to deep vein thrombosis, IS may be experienced by 15% of individuals who are confined to their homes, whereas in the case of disease exacerbation and resultant hospitalisation, IS may affect as many as 27% of them [12]. IS also affects approximately 43–60% of those remaining in institutionalised care [14].

At least two primary and two secondary criteria must effectively be satisfied prior to diagnosing IS. The primary criteria include moderate to severe cognitive deficit and spontaneous muscle cramps. Secondary criteria include skin marks attesting to the individual remaining in the same position over an extensive period, pressure ulcers, dysphagia, aphasia, and urinary incontinence [33]. Of individuals aged 75–81 years who satisfy the IS criteria, 72% are likely to be hospitalised within the following 12 months [34].

Published studies indicate that as far as older adults are concerned, individual lack of self motivation is the second-most common barrier to the pursuit of any physical activity (38.4%), right after feelings of chronic fatigue (51.7%) [35]. As a specific medical condition, IS actually precedes individual immobilisation, which is bound to be the case, given the development of medical conditions caused by a significant mobility deficit. Individual immobilisation may befall anyone, regardless of age. Individual ability to pursue routine activities of daily living tends to drop dramatically, especially in older adults [36], owing mostly to a diversity of medical conditions and/or injuries (Table 1) [37,38,39,40,41,42].

Various studies on older adults have asserted that it tends to occur mostly in hospitalized individuals, as they spend about 83% of their time in bed, whereas in some cases, for example following a hip replacement procedure, this may even be up to 99% [43,44].

Some studies also highlight restriction in individual motor activity of up to 97% [45,46]. Immobilisation is then a natural consequence that affects one in three hospitalised older adults. This also causes a significant reduction in individual physiological reserves (Frailty Syndrome), which is one of the causes of one’s immobilisation [12,47]. A juxtaposition of immobilisation and immobility syndrome is presented in Table 2.

### 3.2. Consequences of Immobilisation and IS

The consequences of immobilisation and IS entail the same bodily systems and structures. The difference actually lies in the development speed of pathologies and dysfunctions limiting the ADLs. Additionally, persistent IS may lead to immobility owing to the development of a diversity of medical conditions originating in the significantly restrained individual motor activity. Apart from general weakness of the body, various physiological disorders in the immobilised patient tend to affect other bodily systems, for example circulatory, respiratory, muscular, skeletal, nervous, urinary, digestive, and even the overall condition of a patient’s skin [48,49].

Such consequences are most likely to be encountered in 5–41% of older adults; muscle cramps being by far the most common effect of individual immobilisation (55%). Approximately 3.4% of older adults, having previously fallen victim to immobilisation, will develop pneumonia, while 1 in 100 individuals will experience inflammation of the urinary tract, deep vein thrombosis, or pressure ulcers, as far as general statistical estimates are concerned [50,51]. The key symptoms accompanying individual immobilisation are summarised in Table 3 [25,48,52,53,54,55,56,57,58,59,60].

### 3.3. Individual Immobilisation and Increased Exposure to Fall Risk

Individual immobilisation is closely associated with hospitalisation in older adults, with the most common causes consisting of femoral neck fracture, Parkinson’s disease, arthritis, and osteoporosis. In the case of ischaemic stroke, this can be about 2–5 days, upon haemorrhagic stroke, up to several weeks, and upon intracerebral haemorrhage, this can be approximately 4 weeks [61]. Exposure to fall risk following hospitalisation has been established to be at its highest within the first 2 weeks following hospital discharge, and tends to stabilise within 3 months or so [62].

Approximately 14% of older adults aged 65 years and over experience a fall within the first month of hospital discharge, whereas 40% of the discharged patients experience a fall within the first 6 months [63,64,65], of whom 50% will most likely sustain an injury [66]. During IS or immobilisation, and immediately afterwards, several symptoms that interact with overall fall risk occur. Muscle strength, muscle function, and sense of balance deteriorate in such individuals [67,68]. In the case of immobilised individuals, their muscle strength deteriorates with each passing week. Within the first week, due to an appreciable loss of muscle mass, muscle strength may drop by 40% [69].

### 3.4. Individual Immobilisation and Additional Fall-Risk Factors

Depleted bone mass and osteoporosis contribute appreciably to an increased risk of sustaining falls, experiencing bone fractures, and requiring recurrent hospitalisations [70]. Regular intake of medications or certain active substances affecting the nervous and circulatory systems may also prove appreciably instrumental in increasing overall fall risk by way of inducing sluggishness, disorientation, dizziness, or poor coordination of movement [71]. Additionally, the cardiorespiratory dysfunction [72], malnutrition, reduced body weight, and attendant postural changes are regarded legitimate risk factors for falls [73,74,75].

**Figure 1:**
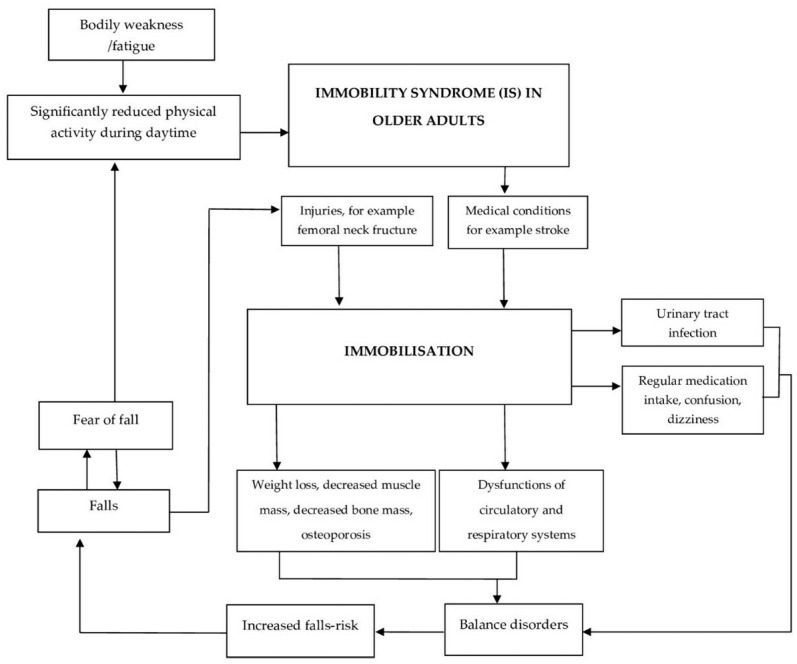
Interactions between older adults affected by IS, individual immobilisation, and falls.

Immobilised individuals often experience a fear of falling as something instilled in them well, especially when said immobilisation is due to having sustained a fall in the first place. This also holds true for other medical conditions, for example, stroke [76,77,78]. Urinary dysfunctions and various infections may also be considered potential consequences (see Figure 1) [79].

### 3.5. Fall Prevention Measures for the Individuals Affected by IS, or Who Have Had Sustained Previous Episodes of Individual Immobilisation

Despite such alarming statistics, a majority of falls can be effectively prevented, and any subsequent readmission to hospital due to any post-fall injuries can also be avoided. It is crucial that health care professionals offer appropriately structured education to patients, as well as attending to implementing effective fall prevention strategies as a legitimate part of the routine hospital discharge procedure [80,81,82]. As far as the individuals affected by IS are concerned, active involvement of their families is very important, as much so as the contribution made by various institutions put in charge of disseminating various pragmatically structured intervention programmes among the patients, specifically aimed at preventing decline in their functional abilities, thus appreciably minimising the overall risk of falling victim to medical conditions that might lead to likely hospitalisation and subsequent immobilisation.

The individuals suffering from a fear of falling should be encouraged to verbalise their feelings, and work on enhancing their own sense of self-reliance in moving about, be that through individual therapy, or structured group activities, or a mix of both approaches. Seniors affected by cognitive impairment should be regularly monitored for cognitive function and offered targeted behavioural therapy.

The key task consists of focusing the work on the patient’s muscular strength and balance, both static and dynamic, which, in conjunction with an appropriately structured dietary intake, would be bound to effectively prevent any detrimental reduction in bone mass.

### 3.6. Immobility Syndrome, Dietary Intake, and Optimised Use of Medications

The right amount of dietary fibre, combined with adequate hydration, fused into an individualised treatment plan, would then translate into a reduced risk of functional dysfunction, as well as addressing individual susceptibility to infections of both the urinary and excretory systems. The actual number and type of medications regularly taken by older individuals should, whenever feasible, be modified to ensure that they do not cause disorientation and dizziness [83,84,85]. Even though older patients often respond quite favourably to any individually tailored education, there is no evidence that such individually targeted education alone would be sufficient to reduce overall fall risk and the actual number of fall episodes, following prior immobilisation directly associated with hospitalisation [86].

### 3.7. Training the IS Patients in Making Use of Effective Fall-Prevention Strategies

In view of the fact that IS, and the period immediately following previous immobilisation of older adults, are invariably associated with general bodily weakness (inclusive of appreciable deterioration in individual muscle strength) [68], and a high fall risk, not only should effective measures be adopted, specifically aimed at implementing effective fall prevention strategies, but the entire approach to this task should be subject to a major rethink, with a view to making it far more comprehensive. It is absolutely vital that seniors should be trained appropriately to be able to cope well on their own (unassisted) after sustaining a fall, provided it has not resulted in any serious injuries [87].

One of the effective ways of coping after an accidental fall is offered by the backward-chaining method [2,88]. This method differs quite distinctly from the conventional methods, as it relies predominantly on the implementation of a reverse sequence of movements by the victim of an accidental fall. Not only does it prove particularly effective at assisting a faller with getting up from the floor entirely on their own, i.e., without the involvement of a third party, but its application is also very user friendly in character.

### 3.8. Unequivocal Terminology Postulated in Diagnosing the Origins of IS or Individual Immobilisation

Individual immobilisation appears now to have become an essential issue within the population of older adults worldwide. As the population of seniors swells, so does the issue at hand. Given its appreciable complexity, it is construed in terms of a diversity of disorders and medical conditions. As no sufficiently coherent and unequivocally verbalised definition has been agreed upon as yet between the academicians actually dealing with it, many terms are still being used interchangeably across the published academic studies, even though individual immobilisation may either originate in, for example individual trauma/surgical intervention, or may well be a result of the appreciable, mainly age-induced, mobility deficit encountered among a vast majority of older adults.

### 3.9. Overall Versatility and Effectiveness of VR Technology Solutions

As is well evidenced across all of the published research, the diverse VR technology solutions boast substantial versatility, which makes them, medically speaking, perfectly viable as easily adaptable therapeutic tools, and are also applicable in patients with specific needs. Their inherent versatility makes it possible to stimulate the patients effectively in many areas of their functional performance, predominantly in the case of those suffering from substantial deficits that usually hamper the pursuit of the activities of daily living, mostly due to excessive fear of falling.

In practical terms, the attending medical personnel may select specific VR solutions believed to be best suited for the task at hand by opting for one of a variety of VR platforms (e.g., Nintendo Wii, Xbox Kinect), through VR goggles (Carl Zeiss), right up to goggles fitted out with hand-held motion controllers (e.g., Oculus Rift S).

## 4. Discussion

To the best of our knowledge, well anchored in a thorough review of the research published to date, our study happens to be the very first one to have focused on comparing individual immobilisation with IS, not only in terms of the applicable epidemiological constraints, but also with respect to relying on the specific insights obtained through hands-on therapeutic management, making use of VR technology, the assessment of changes in individual mobility, the general level of self-reliance in older adults, and their overall exposure to fall risk.

Immobilisation and IS are quite closely related with one another, as older adults tend to spend most of their daytime being inactive, which consequently translates into the development of medical conditions leading directly to subsequent immobilisation. This contributes in due course to the creation of a vicious circle, which in turn poses a significant problem, not only for the older adults themselves, but also for their immediate carers, even though those affected by far the most are the medical personnel and caregiving staff working with these individuals on a daily basis.

Improvements in dynamic balance have been demonstrated in the BBS, TUG and DGI test scores [89,90,91,92,93,94,95], and in static balance in SLS OP and SLS CL, among others [93,94,95,96,97]. Training under VR conditions also translated into improvements in visuospatial function and working memory, as expressed in the TMT B test [96,98]. The feasibility of training with VR when affected by IS was demonstrated by Levy et al., who reported an attenuation of individual fear of falling within 12 weeks of concluding therapy. Obviously enough, this improvement proved crucial for those individuals, as it allowed them to pursue their ADL without excessive fear of falling [99].

The application of VR technology solutions has been corroborated in systematic reviews of studies on older adults [100,101,102]. said the cited studies applied not only to free-living community dwellers [100,101], but also to those affected by immobilisation and IS, e.g., permanent residents of institutional care homes [103]. In terms of individually defined deficits (cognitive function, motor function) and abilities (general motor skills affected by, e.g., severity of IS, dizziness), clinicians may select the therapeutic tools most appropriate for the task by opting for one of a variety of platforms (e.g., Nintendo Wii, Xbox Kinect), through VR goggles (Carl Zeiss), to goggles fitted out with hand-held motion controllers (e.g., Oculus Rift S).

Last but not least, this also accounts for an extra drain on the financial resources of the already overstrained national healthcare systems the world over.

In the present study, we also proposed a specific therapeutic management routine for combating IS, based on our long-term experience in working with the older adults, which accounts for its hands-on application value, in addition to providing a reliable source of credible information for medical personnel working with this particular group of patients. An essential, as well as truly innovative component consists of the introduction of a specifically structured method for coaching older adults in effectively coping after an accidental fall in an exclusively unassisted manner (i.e., with no involvement of third parties), as well as in making use of VR technology for the rehabilitation of immobilised individuals and those affected by IS.

It is the authors’ considered opinion that both the theoretical and the applied aspects, as have been comprehensively addressed in the present study, actually offer appreciable, practical assistance to medical personnel actually involved in care, as well as to national healthcare systems in serious need of having their resources streamlined while achieving a tangible enhancement in overall effectiveness.

## 5. Conclusions

A structured, target-oriented method, innovatively integrated with the selected VR technology solutions, may be comprehensively applied in the rehabilitation of immobilised individuals, as well as those affected by IS. Being principally aimed at improving individual static and dynamic balance, cognitive functions, and at attenuating the fear of falling, it is believed to be of appreciable, practical assistance to all medical personnel involved in therapeutic management.

Last but not least, it also offers a cost-effective way of having this complex issue addressed in any public healthcare system, should it be adopted on a nationwide scale.

## Figures and Tables

**Table 1:** Principal causes of immobilisation in older adults [37,38,39,40,41,42].

**Cardiovascular diseases** Strokes Myocardial infarction **Neurological diseases** Alzheimer’s disease Parkinson’s disease Multiple sclerosis Amyotrophic lateral sclerosis Myasthenia gravis Spinal cord injury	**Respiratory diseases** Chronic obstructive pulmonary disease Pneumonia **Diseases of the osteo-articular system** Osteoporosis Degeneration of the joints (arthritis) Fracture of the femoral neck Injuries

**Table 2:** The differences between immobilisation and IS in older adults.

	Immobilisation	IS in Older Adults
**Age**	Every age range	65 years of age and over
**Limitations in ADL**	Dynamic/sudden	Gradually increasing
**Principal cause**	Disease/injury	Significantly reduced physical activity during daytime
**Place of residence**	Mainly hospital	Mainly institutionalised care/home
**Effect/Cause**	Consequence of hospitalisation/disease(s)	Cause of medical conditions
**As referenced by**	ICD-10	Great Geriatric Problems

IS: immobility syndrome, ADL: activities of daily living, ICD-10: International Classification of Diseases-10.

**Table 3:** Immobilisation symptoms, stratified by their respective systems within the body [25,48,52,53,54,55,56,57,58,59,60].

Bodily System	Symptoms
**Circulatory and respiratory systems**	Fatigue, shortness of breath, palpitations on mild exertion, dizziness, risk of thrombophlebitis, increased risk of pneumonia
**Osteo-articular system**	Decrease in muscle strength, decrease in muscle and bone mass, osteoporosis, postural disorders, muscle cramps, pain
**Nervous system**	Confusion, insomnia, anxiety leading to depression, balance disorders, delirium
**Urinary and excretory systems**	Dehydration, malnutrition, urinary and faecal incontinence, urinary tract infections, excretory infections, constipation
**Skin**	Pressure ulcers

## Data Availability

The datasets generated and/or analysed during the current study are available from the corresponding author upon reasonable request.
